# T3 therapy for hypothyroidism: choosing wisely still requires careful bench to bedside translation

**DOI:** 10.20945/2359-3997000000377

**Published:** 2021-06-29

**Authors:** Jose Miguel Dora, Rafael Selbach Scheffel, Ana Luiza Maia

**Affiliations:** 1 Universidade Federal do Rio Grande do Sul Faculdade de Medicina Hospital de Clínicas de Porto Alegre Porto Alegre RS Brasil Unidade de Tireoide, Hospital de Clínicas de Porto Alegre, Faculdade de Medicina, Universidade Federal do Rio Grande do Sul, Porto Alegre, RS, Brasil

**TO THE EDITOR,**

We thank Zavaleta and cols. (1) for their interest in our work and the opportunity to expand the discussion on Choosing Wisely recommendations for managing thyroid-related conditions (2).

In an average adult, the thyroid gland produces around 80 mcg of thyroxine (T4) and 20 mcg of triiodothyronine (T3) per day to match the body’s metabolic demands. Thus, the activation of the prohormone T4 to the active hormone T3 mainly occurs at peripheral organs, a process fine-tuned by the deiodinases type 1 (D1) and 2 (D2) enzymes accordingly to specific tissue needs. The physiological background and the fact that some patients remain symptomatic regardless of normal thyrotropin (TSH) levels under levothyroxine (LT4) replacement has fuelled the interest in the potential theoretical advantages of adding liothyronine (LT3) to LT4 as a therapeutic strategy. Given that D2 activates approximately 60% of circulating T3 and that some genetic variants of D2, like the D2-Thr92Ala polymorphism, are associated with impaired enzyme activation of T4 into T3, patients homozygotic for the D2-Ala92 genotype might comprise a group that could benefit from combined LT4 + LT3 therapy (3-5). Notwithstanding, despite some preliminary studies had indicated that this might be the case, these findings remain to be confirmed in larger studies before being routinely incorporated in clinical practice.

One should exercise caution when translating bench concepts and preliminary clinical studies to widespread clinical practice, balancing evidence of benefits and harms. In this regard, several studies have attempted to clarify the combined LT4 + LT3 therapy effects. A systematic review that included 9 randomized trials reported beneficial effects of combination LT4 + LT3 therapy only in one trial (6). Subsequently, a meta-analysis of 11 published randomized trials totalizing 1,216 patients showed no benefit of combined LT4 + LT3 therapy on fatigue, bodily pain, anxiety, depression, or general quality of life (7). Of interest, a systematic review that evaluated the pooled prevalence rate for preference of combination therapy over LT4 among hypothyroid patients aware of the combined LT4 + LT3 therapy was 46.2% (95% confidence interval 40.2%, 52.4%) with no difference from chance (P = 0.231) (8).

On the other hand, the potential harms associated with LT3 supplementation can not be underestimated. Since T3 is the active hormone, supraphysiological doses may directly induce thyrotoxicosis, posing risks for serious cardiovascular events (arrhythmias and embolism) and other related adverse effects. Notably, many countries (like Brazil) do not have LT3 formulations commercially available. Thus, in this context, the options to acquire LT3 would rely on formulating LT3 at local pharmacies, a practice that should be highly discouraged given the embodied risks of overdose.

We recognize that knowledge is dynamic and that in the future, we could learn that some subgroups of patients may benefit from LT4 + LT3 therapy for hypothyroidism. If that is the case, we will be glad to revise our position. Until there, we understand that LT4 monotherapy is the most convenient, effective, and safe choice to treat hypothyroidism, maximizing value to our patients.
